# Supplementary figures and images for: Quarantine and the risk of COVID-19 importation
Source: Epidemiol Infect. 2020 Dec 9;148:e298. doi: 10.1017/S0950268820002988 (PMC7783093; doi:10.1017/S0950268820002988)

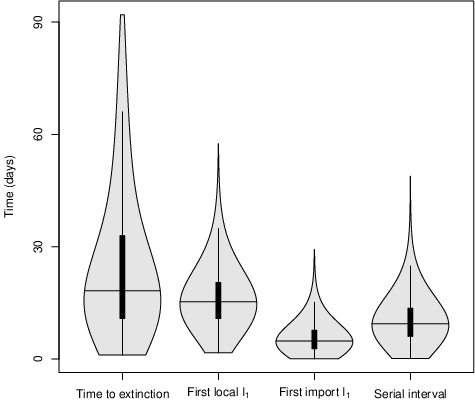

Supplement: Supplementary file 1 [file S0950268820002988sup.zip › S0950268820002988sup002.png]

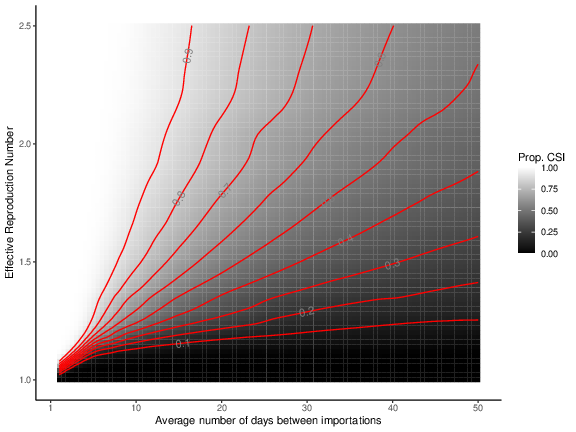

Supplement: Supplementary file 1 [file S0950268820002988sup.zip › S0950268820002988sup003.png]
